# Association of nutritional status and comorbidity with long-term survival among community-dwelling older males

**DOI:** 10.1186/s12877-023-04413-z

**Published:** 2023-10-27

**Authors:** Baicun Hou, Yunjuan Lin, Wangjingyi Zhang, Qiqi Lin, Shengshu Wang, Fansen Meng, Wei Dai, Gangshi Wang

**Affiliations:** 1 Department of Gastroenterology, The Second Medical Center, National Clinical Research Center for Geriatrics Diseases, Chinese PLA General Hospital, Fuxing Road 28#, Haidian district, 100853 Beijing, China; 2 Health Service Department of the Guard Bureau of the Joint Staff Department, 100017 Beijing, China; 3 Medical School of Chinese PLA, Beijing, 100853 China; 4 Institute of Geriatrics, Beijing Key Laboratory of Aging and Geriatrics, National Clinical Research Center for Geriatrics Diseases, Second Medical Center of Chinese PLA General Hospital, 100853 Beijing, China; 5 Office of Information Management, The Second Medical Center, Chinese PLA General Hospital, 100853 Beijing, China

**Keywords:** Long-term mortality, Nutritional status, Comorbidity, Elderly

## Abstract

**Background:**

Estimates of survival in the older can be of benefit in various facets, particularly in medical and individual decision-making. We aim to validate the value of a combination of nutrition status evaluation and comorbidity assessment in predicting long-term survival among community-dwelling older.

**Methods:**

The Charlson Comorbidity Index (CCI) was applied for comprehensive evaluation of comorbidities. Participants were classified into CCI score ≤ 2 and ≥ 3 subgroups. Nutritional status was assessed by using Mini Nutritional Assessment-Short Form (MNA-SF) and Geriatric Nutritional Risk Index (GNRI) evaluations. Mortality rates and survival curves over a 5-year period were compared among subgroups classified by CCI and/or MNA-SF/GNRI evaluations.

**Results:**

A total of 1033 elderly male participants were enrolled in this study, with an average age of 79.44 ± 8.61 years. 108 deceased participants (10.5%) were identified during a follow-up of 5 years. Cox proportional hazards regression analysis showed that age, CCI, MNA-SF and GNRI were independent predictors of 5-year all-cause death in this cohort. Compared to those with normal nutrition status and CCI ≤ 2, the subgroup at risk of malnutrition and CCI ≥ 3 had a significantly higher 5-year all-cause mortality rate (HR = 4.671; 95% CI:2.613–8.351 for MNA-SF and HR = 7.268; 95% CI:3.401–15.530 for GNRI; *P* < 0.001 for both). Receiver operating characteristic curve analysis demonstrated that a combination of either MNA-SF or GNRI with CCI had significantly better performance than CCI, MNA-SF or GNRI alone in predicting all-cause death.

**Conclusion:**

The combination of nutritional assessment (MNA-SF or GNRI) with CCI can significantly improve the predictive accuracy of long-term mortality outcomes among community-dwelling older males.

**Supplementary Information:**

The online version contains supplementary material available at 10.1186/s12877-023-04413-z.

## Introduction

Old people have complex clinical identities and needs, that presented with more comorbidities, cognitive and functional impairments and higher mortality rates than young counterparts [1, 2]. Estimating mortality rates and expected survival among older individuals is valuable for individual decision-making, such as end-of-life decisions or treatment benefits consideration by patients and their family members, which helps to determine the level of care required for those with a fair chance of survival [3]. Age itself is ultimately an important risk factor for death in older adults [4]. Besides, factors related to old age, but not age per se, are reported to be predictive of mortality. These factors included socio-demographic background [5], lifestyles [6, 7], dietary factors [8], life satisfaction [9], metabolic health [10], comorbidities [3] and geriatric syndromes [11], etc.

Comorbidity affects outcomes of the older. The Charlson Comorbidity Index (CCI) is a simple and widely used index for the assessment of comorbidities, and it is the most commonly used and studied for predicting mortality [12]. However, its role to predict long-term clinical outcomes in elderly patients is currently controversial [12–15]. Criticisms include the lack of consideration of disease severity and functional impairment associated with different diseases, as well as the omission of nutritional and social assessments [16]. Similar to comorbidities, nutritional status are confirmed by numerous studies to have a strong association with long-term mortality in the elderly, and it is believed that good nutritional status is significantly correlated with better prognosis [17–19]. Poor nutritional status weakens the body’s immune system and increases the susceptibility to infection-related diseases. While comorbidity and nutritional status are distinct conditions, they are closely related [20–22]. Therefore, a combination of comorbidity assessment with nutritional evaluation may be more effective for predicting mortality. Lee S et al. [23] combined CCI with Geriatric Nutritional Risk Index (GNRI), and found that the GNRI significantly improved long term prognostic predictive accuracy when added to CCI in elderly diffuse large B cell lymphoma (DLBCL) patients. However, the significance of combining CCI with nutritional assessment in predicting long-term mortality among the general elderly population remains unclear.

In this study of a community-dwelling older cohort, we evaluated the nutritional status of enrolled subjects by using Mini Nutritional Assessment-Short Form (MNA-SF) and GNRI, as well as their comorbidities by the CCI score. The associations of comorbidities and malnutrition/nutritional risk with 5-year all-cause mortality were investigated.

## Materials and methods

### Study subjects

This is a retrospective study which is conducted with a cohort of community-dwelling older adults who underwent annual health assessments at the Chinese PLA General Hospital. Detailed clinical data, including survival outcomes, were recorded. We enrolled people with health records between January 2013 and December 2015. This study included male subjects only due to the shorter life expectancy for males compared to females (Available at https://population.un.org/wpp/). Inclusion criteria include male, age 65 to 95 with medical records and relevant laboratory test results. Accidental deaths not caused by diseases were excluded from the study. The Ethics Committee of the General Hospital of Chinese PLA approved this study (Ethics Approval Number: S2020-330-01). As a retrospective statistical analysis based on electronic health records, no individual patients were directly contacted for data collection, and all clinical data involving human participants were treated as confidential and de-identified. The review of medical records by the Ethics Committee of PLA General Hospital was approved, and individual consent for this retrospective analysis was waived.

### Clinical data collection

We reviewed the medical records of all subjects, and collected clinical data at enrollment, including age, height, weight, diagnosis, dietary status, ability to perform daily activities, and mental and psychological status. Mortality data were collected. The time from the initial assessment to death or the last day of follow-up was obtained. Blood test results, including hemoglobin, serum total protein, serum albumin, serum creatinine, lipoprotein, triglyceride, and cholesterol levels, were collected using the medical record management system at the time of enrollment. Blood routine test is performed by XN3000 automatic blood analyzer (Sysmex XN3000, Sysmex Corporation, Kobe, Japan). Biochemical indicators were determined by an electrochemiluminescence immunoassay (Cobas e601, Roche Diagnostics Ltd., Switzerland). Reagents were supplied by equipment manufacturers.

### CCI, MNA-SF and GNRI scores

The CCI scale [24] was used to calculate the comorbidities of the subjects at enrollment. MNA-SF [25] and GNRI [26] scales were used to evaluate the nutritional status of the subjects. The MNA-SF has three classifications: 0–7 points: malnourished; 8–11 points: at risk of malnutrition; or 12–14 points: well-nourished [27]. Participants were subgrouped according to MNA-SF score, into “well-nourished” and “at risk/malnourished”, the latter included those who were at risk of malnutrition and malnourished. The GNRI was calculated from body weight (BW) and serum albumin using the following formula: 14.89 × albumin (g/dl) + 41.7 × (BW/ideal BW). BW/ideal BW was defined as 1 when the patient’s BW exceeded the ideal BW. All patients were categorized into the following four groups according to the GNRI value: no risk (> 98), low risk (92–98), moderate risk (82 to < 92) and major risk (< 82) [26]. Subjects were further divided based on GNRI value, into “no risk” and “nutrition-related risk” subgroups, the latter encompassed low risk, moderate risk and major risk subjects.

### Statistical analysis

The endpoint of the study was death, and the 5-year mortality was defined as the interval from the subjects’ enrollment to the date of all-cause death or the end of the 5-year follow-up period. Continuous variables were expressed as x ± s for variables of normal distribution and median (interquartile range) for variables of skewness distribution. Analysis of variance F test was used for comparison between groups, and Mann-Whitney U test was used for comparison between groups of samples with uneven variance. Categorical data are expressed as numbers and percentages, and the groups were compared using the chi-squared test. Diagnostic performance was assessed by multivariate receiver operating characteristic (ROC) analysis. The area under the receiver operating characteristic curve (AUROC) of the MNA-SF + CCI and GNRI + CCI were compared to the AUROC of the CCI or MNA-SF/GNRI alone using DeLong’s method. The optimal cut-off values of the CCI were identified by ROC analysis using Youden’s index. Survival curves for each group were estimated using the Kaplan–Meier curves and compared by the log-rank test. Cox proportional hazards regression was used to analyze the correlation between variables and 5-year all-cause mortality risk. Two-sided *P* value < 0.05 was considered as statistically significant. GraphPad Prism 7.0 (GraphPad Software, La Jolla, CA, USA,) and SPSS 22.0 (IBM Corp., Armonk, NY, USA) were used for statistical analysis. R-language was used to analyze the additive interaction and multiplicative interaction between CCI and nutritional status. The evaluation indexes of additive interaction included relative excess risk of interaction(RERI),attributable proportion of interaction (API), synergyindex (S). If there is no additive interaction between the two risk factors, then the confidence interval for RERI and API should contain 0, and the confidence interval for S should contain 1.

## Results

### Baseline characteristics

A total of 1221 male subjects (≥ 65 years) were available in this study. Among them, 82 participants were lost to follow-up, and 106 participants had incomplete outcome data. Finally, a total of 1033 elderly men aged 79.44 ± 8.61 years were enrolled. The characteristics of the subjects at the time of enrollment are summarized in Table 1. The median of CCI score of the 1033 study participants was 2 (range, 0–9). 342 (33.1%) subjects presented with CCI score ≥ 3. According to MNA-SF score, 838 (81.1%) subjects were indicated at normal nutrition (well-nourished) in this cohort, while the left 195 (18.9%) subjects were at risk/malnourished. Based on the GNRI score, we identified 945 (95.7%) cases who were at no nutrition-related risk and 88 (4.3%) cases who were at nutrition-related risk. Considering the possibility of attrition bias, we also investigated the relationship between single study variables (CCI, MNA-SF,GNRI) and loss to follow-up. The results showed that these main study variables did not show significant differences between the enrolled and the lost population (Supplementary Table 1).

**Table 1:** General data and clinical features at enrollment

Variable	Total (N = 1033)	Outcomes after 5 years		
		Survival (N = 925)	Death (N = 108)	*P* Value
**Mean ± SD**				
Age(years)	79.44 ± 8.61	78.49 ± 8.39	87.52 ± 5.80	<0.001
BMI(kg/m^2^)	24.43 ± 3.09	24.51 ± 3.10	23.72 ± 2.93	0.315
Total protein (g/L)	71.88 ± 4.92	72.00 ± 4.76	70.84 ± 6.08	0.007
Albumin (g/L)	45.16 ± 3.41	45.38 ± 3.18	43.32 ± 4.61	<0.001
Hemoglobin (g/L)	142.48 ± 15.94	142.99 ± 15.20	138.09 ± 20.87	<0.001
Low density lipoprotein (mmol/L)	2.75 ± 0.82	2.76 ± 0.83	2.68 ± 0.76	0.379
Total cholesterol (mmol/L)	4.38 ± 0.93	4.38 ± 0.93	4.35 ± 0.91	0.433
Triglyceride (mmol/L)	1.42 ± 0.80	1.43 ± 0.81	1.32 ± 0.69	0.432
Alkaline phosphatase (U/L)	63.08 ± 16.71	62.95 ± 16.70	64.24 ± 16.87	0.490
Uric acid (µmol/L)	350.33 ± 71.27	349.63 ± 71.34	356.31 ± 70.75	0.855
Serum iron (µmol/L)	20.25 ± 6.00	20.02 ± 6.02	19.33 ± 6.21	0.681
Calcium (mmol/L)	2.32 ± 0.09	2.32 ± 0.09	2.31 ± 0.10	0.393
Phosphorus (mmol/L)	1.10 ± 0.15	1.10 ± 0.15	1.10 ± 0.15	0.497
**Median(P25, P75)**				
Creatinine (µmol/L)	85.0(77.0,97.0)	85.0(77.0,96.0)	87.0(77.8,101.5)	0.206
Blood urea nitrogen(BUN) (mmol/L)	5.8(5.0,7.0)	5.8(4.9,6.9)	5.9(5.1,7.4)	0.180
Blood glucose (mmol/L)	5.58(5.21,6.17)	5.59(5.22,6.18)	5.55(5.10,6.11)	0.344
γ- glutamyl transpeptidase (U/L)	23.0(17.0,33.0)	23.0(17.0,33.0)	22.5(15.3,34.3)	0.612
Lactate dehydrogenase (U/L)	172.5(154.0,194.0)	173.0(154.0,194.0)	169.0(152.3,197.8)	0.808
Alanine aminotransferase (U/L)	16.5(12.1,22.0)	17.0(12.4,22.0)	16.0(12.0,20.3)	0.098
Aspartate transaminase (U/L)	19.0(16.0,22.0)	19.0(16.0,22.0)	18.1(16.4,22.4)	0.745
Total bilirubin (µmol/L)	11.80(9.20,15.08)	11.80(9.20,15.03)	12.00(9.18,15.40)	0.970
BUN/albumin(BAR)(mg/g)	3.6(3.0,4.4)	3.6(3.0,4.3)	3.9 (3.2,5.0)	0.007
CCI	2.0(1.0,3.0)	2.0(1.0,3.0)	3.0(2.0,4.0)	< 0.001
MNA-SF	13.0(12.0,13.0)	13.0(12.0,13.0)	12.0(11.0,13.0)	0.001
GNRI	112.6(107.7,117.5)	113.0(108.4,117.7)	108.4(102.5,113.2)	< 0.001
** N(%)**				
CCI	1033			< 0.001
≤2	691(66.9)	646(69.8)	45(41.7)	
≥3	342(33.1)	279(30.2)	63(58.3)	
MNA-SF	1033	925	108	0.001
12–14 (Normal nutrition)	838(81.1)	763(82.5)	75(69.4)	
0–11 (At risk/malnourished)	195(18.9)	162(17.5)	33(30.6)	
GNRI	987	879	108	< 0.001
>98 (No Nutrition-related risk)	945(95.7)	850(96.7)	95(88.0)	
≤ 98 (Nutrition-related risk)	42(4.3)	29(3.3)	13(12.0)	

### Follow-up and survival analysis

During a follow-up of 5 years, all-cause mortality was ascertained and 108 deceased participants (10.5%) were identified. A comparison of baseline characteristics between the survival and dead groups were performed. Univariable predictors of mortality included age, serum total protein, albumin, hemoglobin, BUN/albumin (BAR), CCI, MNA-SF and GNRI (Table 1). When conducting multivariate Cox proportional hazards analysis, we merged certain anthropometric parameters or composite indicators that reflect organ function. These parameters included age, serum lipids (total cholesterol and triglycerides), kidney function (creatinine), liver enzymes indicating hepatocyte damage (alanine aminotransferase), enzymes indicating cholestasis (total bilirubin), blood glucose, uric acid, serum iron, nutritional status (MNA-SF and GNRI), comorbidities (CCI) and BUN/albumin (BAR). The results of the multivariate analysis showed that age, CCI and nutritional status were independent predictors of 5-year all-cause mortality. Malnutrition (at risk/malnourished versus no nutrition-related risk) was associated with significantly increased risk for mortality as assessed by MNA-SF (HR = 0.859; 95%CI:0.742–0.995; *P* = 0.043) (Table 2, Model 1) or GNRI (HR = 0.981; 95%CI:0.964–0.998; *P* = 0.033) (Table 2, Model 2).

**Table 2:** Multivariate Cox proportional hazards analysis of 5-year all-cause mortality

Model	Variable	HR(95%CI)	*P* Value
**Model 1** ^a^	age	1.180(1.127–1.236)	< 0.001
	CCI	1.269(1.109–1.452)	0.001
	MNA-SF	0.859(0.742–0.995)	0.043
**Model 2** ^b^	age	1.167(1.114–1.222)	< 0.001
	CCI	1.247(1.089–1.427)	0.001
	GNRI	0.981(0.964–0.998)	0.033

a Adjusted for age, triglyceride, total cholesterol, blood glucose, serum iron, creatinine, uric acid, total bilirubin, alanine aminotransferase, blood urea nitrogen (BUN)/albumin (BAR), CCI, and MNA-SF

b Adjusted for age, triglyceride, total cholesterol, blood glucose, serum iron, creatinine, uric acid, total bilirubin, alanine aminotransferase, blood urea nitrogen (BUN)/albumin (BAR), CCI, and GNRI

### Prognostic stratification based on CCI and nutritional status

The ROC analysis showed that the optimal cut-off value of CCI was 2.5 (sensitivity 58.3%, specificity 69.8%). Participants were then divided into two subgroups for further analysis: CCI ≤ 2 (low CCI score) and CCI ≥ 3 (high CCI score). Based on the combination of CCI and MNA-SF, all subjects were classified into four subgroups: CCI ≤ 2 with normal nutrition (L-NN); CCI ≤ 2 with at risk/malnourished (L-ARM); CCI ≥ 3 with normal nutrition (H-NN); CCI ≥ 3 with at risk/malnourished (H-ARM). The 5-year mortality rates of these groups were 5.3%, 12.3%, 16.9%, and 23.5%, respectively. Similarly, according to the combination of CCI and GNRI, all subjects were divided into four groups: CCI ≤ 2 with no nutrition-related risk (L-NNR); CCI ≤ 2 with nutrition-related risk (L-NR); CCI ≥ 3 with No nutrition-related risk (H-NNR); CCI ≥ 3 with nutrition-related risk (H-NR). The 5-year mortality rates for each group were 6.4%, 25.0%, 17.4%, and 36.4%, respectively.

**Fig. 1:**
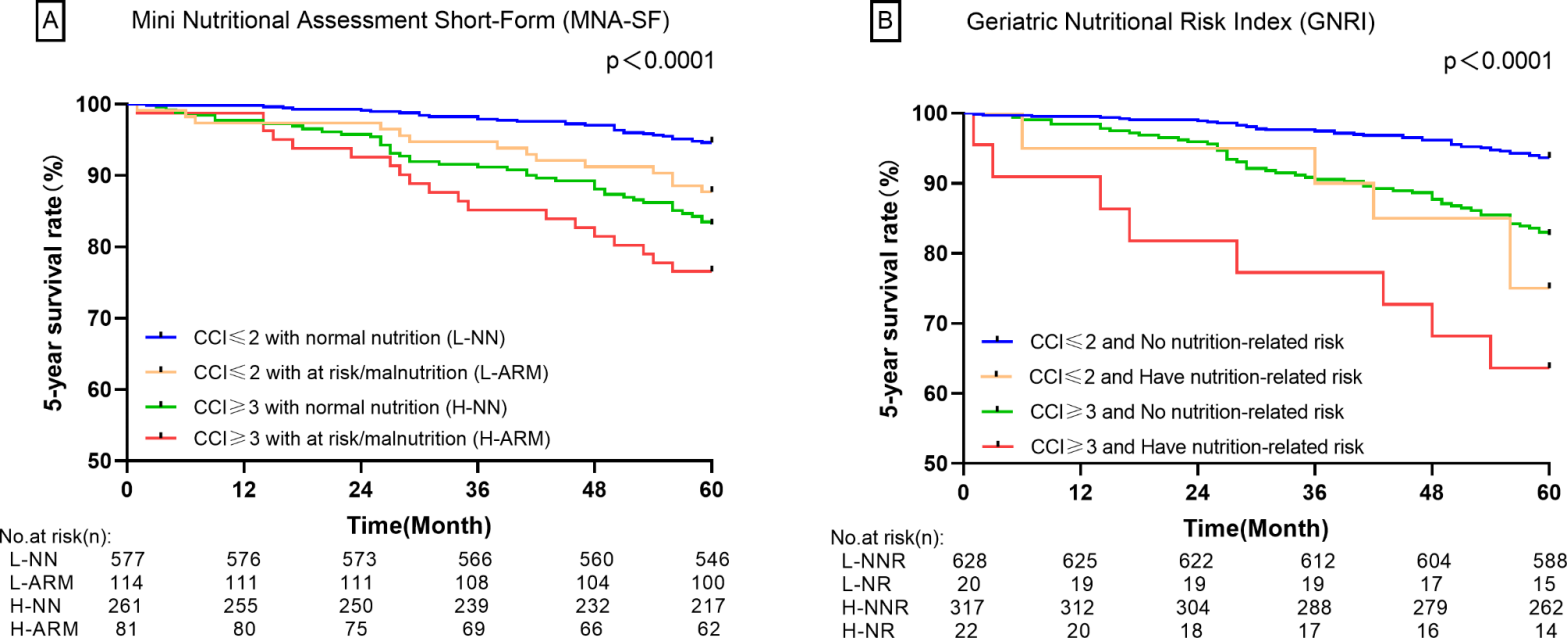
5-year survival curves based on CCI and nutritional status

In the Cox proportional hazards regression analyses, L-NN and L-NNR were used as the reference group. Compared with subgroup in normal nutrition status and CCI ≤ 2, the 5-year all-cause mortality rate was significantly increased in those at risk of malnutrition and CCI ≥ 3 (HR = 4.671; 95% CI:2.613–8.351 for MNA-SF and HR = 7.268; 95% CI:3.401–15.530 for GNRI; *P* < 0.001 for both) (Table 3). The Kaplan-Meier curves (Fig. 1) indicate significant differences in survival between the CCI ≤ 2 with normal nutrition group and the CCI ≥ 3 with malnutrition group. Considering the possible interaction between CCI and nutritional status on long-term survival, the additive and multiplicative models were applied to analyze the interaction effect of them. The results showed that although the risk of malnutrition increased with higher comorbidities scores (Supplementary Table 2), there was no additive interaction and multiplicative interaction between CCI and MNA-SF/GNRI (Supplementary Tables 3 and Supplementary Table 4).

**Table 3:** Cox proportional hazards analysis of 5-year all-cause mortality in older adults with different CCI and nutritional status

CCI	MNA-SF	Nutritional status	Group	Mortality(%)	HR(95%CI)^a^	*P* Value
≤ 2	12–14	Normal nutrition	L-NN	5.4	1[Reference]	
	0–11	At risk/malnourished	L-ARM	12.3	2.379(1.266–4.472)	0.007
≥ 3	12–14	Normal nutrition	H-NN	17.2	3.434(2.173–5.426)	< 0.001
	0–11	At risk/malnourished	H-ARM	22.5	4.671(2.613–8.351)	< 0.001
**CCI**	**GRNI**	**Nutritional status**	**Group**	**Mortality(%)**	**HR(95%CI)** ^**b**^	***P*** **Value**
≤ 2	>98	No Nutrition-related risk	L-NNR	6.4	1[Reference]	
	≤ 98	Nutrition-related risk	L-NR	25.0	4.259(1.681–10.791)	0.002
≥ 3	>98	No Nutrition-related risk	H-NNR	17.4	2.906(1.934–4.367)	< 0.001
	≤ 98	Nutrition-related risk	H-NR	36.4	7.268(3.401–15.530)	< 0.001

a Adjusted for age, triglyceride, total cholesterol, blood glucose, serum iron, creatinine, uric acid, total bilirubin, alanine aminotransferase, blood urea nitrogen (BUN)/albumin (BAR), CCI, and MNA-SF

b Adjusted for age, triglyceride, total cholesterol, blood glucose, serum iron, creatinine, uric acid, total bilirubin, alanine aminotransferase, blood urea nitrogen (BUN)/albumin (BAR), CCI, and GNRI

### Predictive value of CCI and nutritional status in all-cause mortality

**Fig. 2:**
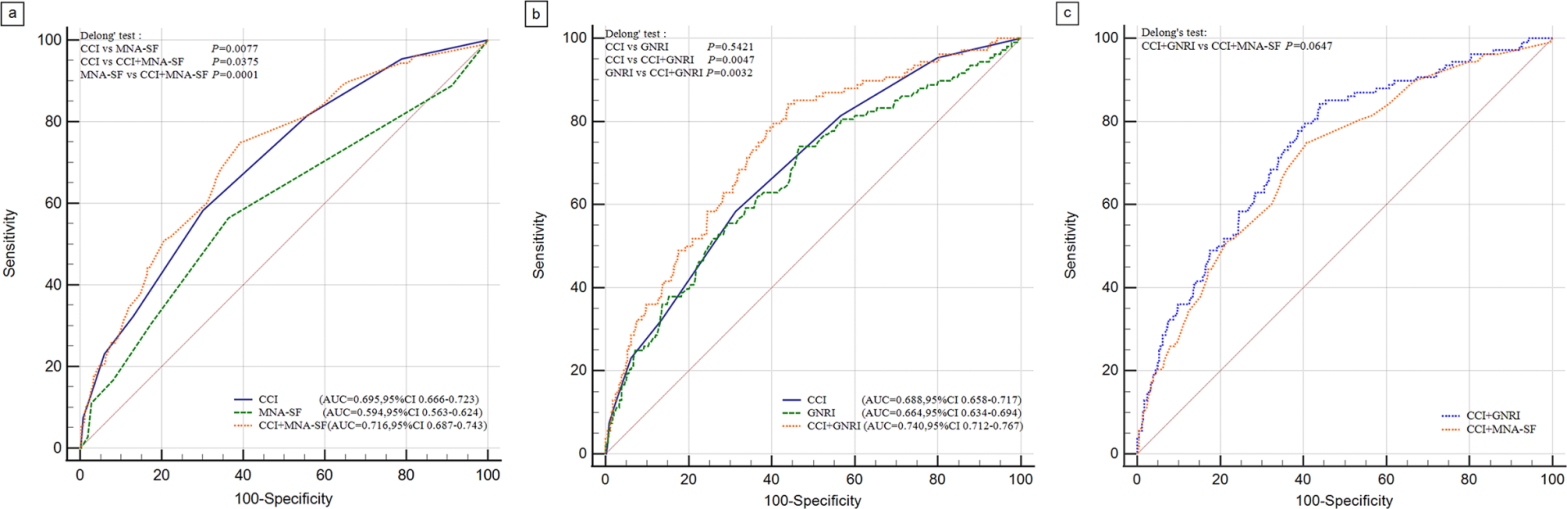
Receiver operating characteristic curves showing the performance of CCI and nutritional status for predicting 5-year mortality. **a** ROC curves of CCI,MNA-SF and CCI + MNA-SF. **b** ROC curves of CCI,GNRI and CCI + GNRI. **c** ROC curves of CCI + GNRI and CCI + MNA

We performed ROC curve analysis to compare the predictive accuracy of different measures. As shown in Fig. 2, when compared with single index, the combination of CCI and MNA-SF showed significantly better performance (AUC, 0.716; 95% CI: 0.687–0.743) than CCI alone (AUC, 0.695; 95% CI: 0.666–0.723), and MNA-SF alone (AUC, 0.594; 95% CI: 0.563–0.624) in predicting all-cause death (all DeLong’ test *P* for difference in AUC < 0.05) (Fig. 2a). The Z value for each pairwise AUC was 2.666(CCI vs MNA-SF),2.081(CCI vs CCI + MNA-SF),3.897(MNA-SF vs CCI + MNA-SF). The standard error for each pairwise AUC was 0.0379(CCI vs MNA-SF),0.00998(CCI vs CCI + MNA-SF),0.0313(MNA-SF vs CCI + MNA-SF) (Fig. 2a). Similarly, the combination of CCI and GNRI showed significantly better performance (AUC, 0.740; 95% CI: 0.712–0.767) than CCI alone (AUC, 0.688; 95% CI: 0.658–0.717), and GNRI alone (AUC, 0.664; 95% CI: 0.634–0.694) (all DeLong’ test *P* for difference in AUC < 0.005), although there is no difference in the prediction accuracy between CCI and GNRI (*P* = 0.5421) (Fig. 2b). The Z value for each pairwise AUC was 0.610(CCI vs GNRI),2.825(CCI vs CCI + GNRI),2.952(GNRI vs CCI + GNRI).The standard error for each pairwise AUC was 0.0389(CCI vs GNRI),0.0184(CCI vs CCI + GNRI),0.0256(GNRI vs CCI + GNRI) (Fig. 2b). When the combination of CCI and GNRI is compared with the combination of CCI and MNA-SF, there is no obvious difference in their performance (*P* = 0.0647) (Fig. 2c). The Z value was 1.847 and the standard error was 0.0174 for CCI + GNRI vs CCI + MNA-SF (Fig. 2c).

## Discussion

Numerous studies have shown that the mortality of community-dwelling older adults is influenced to multiple factors, including comorbidities [28], community activities [29], cognitive function [30], nutritional status [31, 32], and others. In this study, the results of Cox proportional risk regression indicated that age, CCI, MNA-SF or GNRI were independent factors affecting the 5-year all-cause mortality of the elderly. While chronological age cannot be changed, we can make a difference in geriatric practice by controlling comorbidities and adjusting nutrition to benefit the older. The association of comorbidities with mortality in the elderly were consistently demonstrated in many studies [33–35]. CCI has been widely used to predict short-term clinical outcomes and been validated in various populations [36–39]. Some studies also found that CCI could help to predict long-term mortality in different clinical populations, including medical, surgical, intensive care unit, trauma, and cancer patients [40–44]. In our study, the CCI score proved to be an independent predictor of 5-year mortality (HR = 1.269, 95%CI: 1.109–1.452, *P* = 0.001), suggesting that CCI has predictive value for long-term mortality in the general elderly population. However, the role of CCI for long-term mortality prediction in the older is still controversial. Gianluca Testa et al. found that CCI does not predict long-term mortality in elderly patients with chronic heart failure [14], while Frenkel et al. indicated that the CCI independently predicts 3-month, 1-year, and 5-year mortality in acutely ill hospitalized elderly adults [3]. The fact that CCI did not include risk factors, such as functional assessments, social and nutritional status, might decrease its predictive value. Therefore, some studies have attempted to combine CCI with other indicators to compensate for its deficiency.

Malnourished older adults are at high risk of mortality. Nutritional status has been reported as a predictor of complications and outcomes of various entities [45–47]. MNA-SF [25] and GNRI [26] are commonly used nutritional screening tools for the elderly. Both of them are helpful to predict the long-term prognosis of elderly patients and a poor nutritional status is associated with an elevated risk of all-cause mortality [48–51]. MNA-SF involves subjective questions and GNRI relies on objective indicators, making them complementary in assessing nutritional status. Therefore, we used these two methods to assess nutritional status in the same population to compare the difference of these two nutritional assessment methods in predicting long-term mortality in the elderly. As expected, we found that both MNA-SF and GNRI were independent predictors of 5-year mortality in this cohort, in which the elderly who exhibited lower MNA-SF or GNRI score had a higher risk of death within 5 years. It has been reported that MNA-SF has a greater tendency to classify patients as malnourished than GNRI does [52, 53]. This was corroborated by the results in this study, where the diagnostic rates of (being at risk of) malnutrition for MNA-SF and GNRI were 18.9% and 4.3%, respectively.

To our knowledge, the present study is the first to combine nutritional screening tools and CCI to predict long-term survival in community-dwelling older adults. One of the significant highlights of this study is that all subjects were divided into four groups based on their malnutrition risk and CCI scores. The 5-year mortality risk was significantly higher for older adults with CCI ≥ 3 and poor nutrition (both MNA-SF and GNRI) compared to those with CCI ≤ 2 and normal nutrition. This suggests a promising role of this combination of evaluation in identifying high risk individuals with poor long-term outcomes in community-dwelling older adults, and consequencely, effective interventions on these subgroups will improve their outcomes. Besides CCI scores, other indicators combined with nutritional status can also predict mortality outcomes in older adults. A cohort study in Singapore involving 2804 community-dwelling adults discovered that poor nutrition combined with prefrailty/frailty was associated with substantially increased prevalence and incidence of poor functional and mortality outcomes [54]. Handgrip strength, an objective marker of frailty, has been shown to independently predict adverse health outcomes and mortality in older populations and different clinical settings [55]. In the future, we expect that more easy-to-get indicators will be reported for predicting mortality in the elderly population.

It should be noted that although there is a significant relationship in the hazard ratio between higher CCI score and mortality in older adults [16], the optimal cut-off value for CCI is still unclear. In our study, based on ROC analysis, we defined high-risk comorbidities as having a CCI of ≥ 3 and low-risk comorbidities as having a CCI of ≤ 2, which is consistent with previous studies [21, 56]. Nonetheless, further research is needed to determine the optimal cut-off value for CCI.

There are several limitations to consider in this study. Firstly, this was a retrospective, single-center study involving community-dwelling older adults. External validation through cohort studies conducted in different settings is necessary to confirm the robustness of our findings. Secondly, although the CCI, MNA-SF and GNRI are continuous variables, they were transformed into categorical variables when combined. Therefore, further clarification is needed to determine the optimal cut-off values for CCI, MNA-SF and GNRI. Thirdly, the study population consisted of elderly male living in the communities with high-level healthcare services, which may not fully represent the general elderly population. However, given the average life expectancy in China being 74.7 years for males and 80.5 years for females [57], studing factors related to the long-term survival in elderly male subgroups can still be meaningful. Lastly, more detailed information about prognostic outcomes might be provided if periodical assessment of CCI and nutrition scores were obtained.

In summary, our findings suggest that CCI, MNA-SF and GNRI are independent factors affecting 5-year all-cause mortality in community-dwelling older males. Combining nutritional assessment (using either MNA-SF or GNRI) with CCI significantly improves the predictive accuracy of long-term mortality outcomes in this cohort. Interventional studies that investigate the improvement of nutritional status could potentially lead to favorable mortality outcomes in the elderly. Multi-centered, large scale and prospective studies are needed to validate this conclusion.

## Conclusion

The combination of nutritional assessment (MNA-SF or GNRI) with CCI can significantly improve the predictive accuracy of long-term mortality outcomes among community-dwelling older males.

### Electronic supplementary material

Below is the link to the electronic supplementary material.

Supplementary Material 1

## Data Availability

The datasets generated for this research are available from the corresponding author on reasonable request.

## Abbreviations

AUROC: area under the receiver operating characteristic curve
BAR: BUN/albumin
BUN: blood urea nitrogen
BW: body weight
CCI: charlson comorbidity index
CI: confidence interval
DLBCL: diffuse large B cell lymphoma
GNRI: geriatric nutritional risk index
MNA-SF: mini nutritional assessment-short form
OR: odds ratio
ROC: receiver operating characteristic
