# Supplementary material for: Association of nutritional status and comorbidity with long-term survival among community-dwelling older males
Source: BMC Geriatr. 2023 Oct 27;23:697. doi: 10.1186/s12877-023-04413-z (PMC10605511; doi:10.1186/s12877-023-04413-z)
Supplement: Supplementary file 1 — Supplementary Material 1 [file 12877_2023_4413_MOESM1_ESM.doc]

**Supplementary Table 1.** **The relationship between single study variables**

**( CCI, MNA-SF,GNRI) and loss to follow-up**

| **Variable**  **Median(P25，P75)** | **Enrolled population (N=1033)** | **Loss to follow up**  **(N=82)** | ***P* Value** |
| --- | --- | --- | --- |
| CCI | 2.0(1.0,3.0) | 2.0(1.0,3.0) | 0.665 |
| MNA-SF | 13.0(12.0,13.0) | 13.0(12.0,13.0) | 0.091 |
| GNRI | 112.5(107.7,117.5) | 112.6(109.0,117.7) | 0.957 |

**Supplementary Table 2. The relationship between CCI and nutritional status**

| **Variable** | **CCI≤2 N(%)** | **CCI≥3 N(%)** | ***P* Value** |
| --- | --- | --- | --- |
| MNA-SF |  |  | 0.005 |
| 12-14(Normal nutrition) | 577(68.9) | 261(31.1) |  |
| 0-11(At risk/malnourished) | 114(58.5) | 81(41.5) |  |
| GNRI |  |  | 0.012 |
| ＞98(No Nutrition-related risk) | 628(66.5) | 317(33.5) |  |
| ≤98(Nutrition-related risk) | 20(47.6) | 22(52.4) |  |

**Supplementary Table 3.**

**The additive interaction between CCI and nutritional status**

| **CCI and nutritional status** | **Index** | **Value** | **95%CI** |
| --- | --- | --- | --- |
|  | RERI | 0.360 | -2.954-3.676 |
| CCI and MNA-SF | API | 0.067 | -0.521-0.655 |
|  | S | 1.089 | 0.500-2.373 |
|  | RERI | 1.414 | -7.455-10.283 |
| CCI and GNRI | API | 0.168 | -0.772-1.108 |
|  | S | 1.236 | 0.338-4.528 |

**Supplementary Table 4.**

**The multiplicative interaction between CCI and** **nutritional status**

| **Variable** | **β** | **S.E.** | **Wald** | ***P* Value** | **OR(95%CI)** |
| --- | --- | --- | --- | --- | --- |
| CCI≥3  (age-adjusted) | 1.273  0.658 | 0.248  0.266 | 26.379  6.149 | 0.000  0.013 | 3.571(2.197-5.805)  1.932(1.148-3.250) |
| MNA-SF：0-11(At risk/malnourished)  (age-adjusted) | 0.903  0.402 | 0.340  0.362 | 7.051  1.230 | 0.008  0.267 | 2.466(1.267-4.800)  1.494(0.735-3.039) |
| CCI≥3 and MNA-SF：0-11  (age-adjusted) | -0.490  -0.192 | 0.460  0.487 | 1.132  0.156 | 0.287  0.693 | 0.613(0.249-1.510)  0.825(0.317-2.144) |
| CCI≥3  (age-adjusted) | 1.127  0.595 | 0.221  0.235 | 26.073  6.390 | 0.000  0.011 | 3.086(2.002-4.756)  1.813(1.143-2.876) |
| GNRI≤98(Nutrition-related risk)  (age-adjusted) | 1.589  0.912 | 0.542  0.608 | 8.609  2.252 | 0.003  0.133 | 4.900(1.695-14.166)  2.490(0.756-8.194) |
| CCI≥3 and GNRI≤98  (age-adjusted) | -0.588  -0.387 | 0.715  0.781 | 0.675  0.245 | 0.411  0.620 | 0.556(0.137- 2.258)  0.679(0.147-3.140) |
